# Effects of Transglutaminase and Heat Treatment on the Structure and Gelation Properties of Camel Casein Protein

**DOI:** 10.3390/foods14091644

**Published:** 2025-05-07

**Authors:** Qing Niu, Enhui Liu, Chenkun Huo, Fei Zhang, Ruiqi He, Jie Yang, Zhongkai Zhao

**Affiliations:** 1 College of Smart Agriculture (Research Institute), Xinjiang University, Urumqi 830017, China; shi677777@163.com (Q.N.); liuenhui_nori@126.com (E.L.); 18230115768@163.com (C.H.); zf13856123842@163.com (F.Z.); ruiqi_h@163.com (R.H.); 2 Xinjiang Key Laboratory of Biological Resources Genetic Engineering, Urumqi 830017, China; yangjie234@xju.edu.cn; 3 Xinjiang Camel Industry Engineering Technology Research Center, Urumqi 830046, China

**Keywords:** transglutaminase, camel casein protein, enzymatic crosslinking, heat treatment, gelation properties

## Abstract

This study investigated the effects of transglutaminase (TGase) content (0%, 0.5%, 1%, 1.5%) and heat treatment (25 °C, 70 °C, 80 °C, 90 °C) on the structure and gel properties of camel casein protein. The results indicate that a TGase concentration of 0.5% combined with a heat treatment of 90 °C in SDS-PAGE facilitates the aggregation and crosslinking of protein molecules to form polymers, with the degree of crosslinking increasing alongside the TGase concentration. In FTIR, the treatment with TGase and heat resulted in a shift of the absorption peak of the amide I band, indicating a transition of the secondary structure from a loose to an ordered configuration. Additionally, surface hydrophobicity and heat enthalpy values were significantly increased, while the thermal transition temperature of casein gradually decreased. Following TGase binding and heat treatment, casein protein molecules formed a network structure characterized by small pore sizes and close crosslinking. Rheological analysis revealed that 0.5% TGase treatment significantly lowered the gel formation point of casein, promoted gelation, and effectively enhanced the mechanical properties and water-holding capacity of the casein gels. These findings provide theoretical reference for the development of camel protein modification and gel products.

## 1. Introduction

Camel milk, a unique dairy product from Xinjiang, is rich in bioactive substances such as lysozyme, lactoferrin, and active peptides. It exhibits antibacterial, anti-inflammatory, and antioxidant properties [1,2,3] and has been shown to assist in alleviating conditions such as diabetes, hypertension, and liver and kidney injuries, and it has anti-tumor effects [4,5,6,7,8]. Compared to cow’s milk, camel milk contains lower levels of saturated fatty acids and higher levels of unsaturated fatty acids, which enhances its nutritional value. Moreover, it demonstrates superior antioxidant properties and angiotensin-converting enzyme (ACE) inhibition during simulated gastrointestinal digestion, thus providing greater health benefits [9]. The composition of camel milk closely resembles that of human breast milk, offering a high digestion and absorption rate along with a low allergy rate, making it particularly suitable for the nutritional needs of infants and young children [10]. In recent years, consumer awareness of the health and the nutritional and functional benefits of camel milk has increased, leading to a growing demand for camel milk products. Casein, the primary structural protein in milk, constitutes approximately 80% of the protein in cow’s milk, whereas in camel milk, casein accounts for 50–80% of the total protein content, predominantly comprising β-casein (65%) and αs1-casein (21%) [11]. The proportions of αs2-casein and κ-casein are relatively low, at 10% and 3.5%, respectively [12]. The differences in casein composition and content between camel milk and cow’s milk not only affect their nutritional value but also determine the functional properties of the casein components. Consequently, their contributions to product processing, organization, and morphology significantly differ from those of cow’s milk.

Casein gel represents a significant area of casein processing and application. Due to its high safety, processing plasticity, and viscoelasticity, casein gel is widely utilized in various aspects of food processing. It serves as a natural food gel that enhances the texture and stability of dairy products, controls water migration, and maintains product shape, thereby improving product quality and stability while optimizing taste [13]. In addition, casein gels serve as effective delivery carriers for bioactive ingredients, such as lactoferrin and immunoglobulin, as well as for drugs. They enhance the stability and bioavailability of these compounds while allowing for controlled release rates, thereby facilitating precise nutritional supplementation [14].

Protein gels are typically produced through heat induction. As the temperature increases, the protein structures undergo stretching and denaturation, leading to molecular aggregation. Upon reaching the gel formation point—the lowest temperature at which gelation occurs—crosslinking between protein molecules establishes a primary network. During the subsequent cooling process, numerous hydrogen bonds are formed, ultimately resulting in a three-dimensional network structure [15]. The average diameter of casein micelles in camel milk ranges from 240 to 280 nm, which is approximately twice bigger than that of casein micelles in cow’s milk (100–140 nm). This larger-size feature makes it more challenging for camel milk to form a dense gel structure [16,17]. Furthermore, the κ-casein content in cow’s milk is relatively high, allowing it to interact with the other three types of caseins through hydrophobic interactions. This interaction helps stabilize the casein micelles by covering their surface. In contrast, the lower κ-casein content in camel milk results in a comparatively weaker stabilizing effect on the micelles [18]. Consequently, enhancing the gelling properties of camel milk proteins through simple and environmentally friendly modification techniques—such as enzymatic treatment or heat treatment—has becoming a focal point of research on camel milk proteins.

Transglutaminase (TGase, EC 2.3.2.13) is an acyltransferase that catalyzes the acyl transfer reaction between the γ-carboxamide group of glutamine residues and the ε-amino group of lysine residues in proteins, resulting in the formation of covalent crosslinks known as ε-(γ-glutamyl)-lysine isopeptide bonds [19]. This enzyme finds extensive applications in various fields, including seafood, surimi products, meat products, pasta, dairy products, and baked goods. Through mild enzymatic reactions, it can significantly enhance product hardness, elasticity, viscosity, thermal stability, and water retention capacity [20]. Previous research indicates that the introduction of transglutaminase or functional amino acids can promote the aggregation of myofibrillar proteins in meat, thereby improving protein gelation while reducing salt usage [21,22]. In dairy products specifically, TGase catalysis has been shown to markedly increase the apparent viscosity of yogurt and improve both the water-holding capacity and dehydration resistance [23]. Furthermore, Chen et al. discovered that TGase facilitates the formation of covalent bonds between milk proteins, which enhances structural integrity and textural stability in dairy products [24]. However, there remains a paucity of research regarding the application of TGase in camel milk products and milk protein processing.

The gelation properties of proteins are crucial in production and processing. Based on the induction method, protein gelation can be categorized into thermally induced and non-thermally induced gelation. During the process of protein gelation, both the thermal treatment and the enzymatic treatment play key roles. However, there has yet to be a systematic investigation into the combined effects of thermal treatment and enzymatic treatment on the gelation of camel milk casein proteins. Therefore, this study aims to explore the impact of transglutaminase in combination with thermal treatment on the structure and gel characteristics of camel milk casein proteins. The findings will provide new insights for research on camel milk casein protein properties, as well as for the development of camel milk functional gel products.

## 2. Materials and Methods

### 2.1. Materials

The raw camel milk was collected from the Bactrian camel breeding in Fukang city. Microbial transglutaminase (TGase, EC 2.3.2.13) was purchased from Solarbio Co. Ltd. (Beijing, China). Tris base, β-mercaptoethanol, and glycine were purchased from Sigma–Aldrich, Inc. (St. Louis, MO, USA). Other chemicals and reagents were of analytical grade.

### 2.2. Preparation of Camel Casein Protein Samples

Fresh camel milk was centrifuged at 4 °C and 10,000 r/min for 10 min, and the upper layer was skimmed to obtain defatted milk. The pH of the defatted camel milk was adjusted to 4.2 (isoelectric point of camel casein protein) by using 10% glacial acetic acid solution, then placed in a refrigerator at 4 °C for 3 h to collect the protein precipitate. Subsequently, the settled samples were removed and subjected to centrifugation at 8000 rpm for 10 min at 4 °C. The lower casein precipitate was collected and followed by washing three times with distilled water, then lyophilized to obtain sample powder with 92% purity, as measured by the Kjeldahl method (N % × 6.40). Each sample was divided into three parts after lyophilization for TGase, heat treatment and TGase, and heat treatment.

Simply, for heat treatment investigations, camel casein protein samples were incubated at 25, 70, 80, and 90 °C for 30 min. Subsequently, transglutaminase (TG) enzyme was added at concentrations of 0%, 0.5%, 1%, and 1.5% under optimal enzymatic processing conditions (temperature: 50 °C, pH: 7.0). The mixture was inactivated by heating at 90 °C for 5 min. Finally, all samples were freeze-dried, then stored at −18 °C for further analysis.

### 2.3. Sodium Dodecyl Sulfate Polyacrylamide Gel Electrophoresis (SDS–PAGE)

The molecular weight distribution was investigated using the SDS-PAGE electrophoresis system (model AE-6450, ATTO Co., Tokyo, Japan). Both 12.5% acrylamide separation adhesive and 5% acrylamide concentrating adhesive were employed for the experiment. The sample volume was set at 10 µL, and the electrophoresis conditions consisted of 90 V constant-voltage electrophoresis for 20 min, followed by 120 V constant-voltage electrophoresis for 2 h. The molecular weights of the protein bands were compared with those of low-molecular-weight standards (10–180 kDa, Solarbio, Beijing, China) [25].

### 2.4. Fourier Transform Infrared Spectroscopy (FTIR)

Under dry conditions, 1 mg of the sample was thoroughly ground with potassium bromide, using an agate mortar to obtain a uniform powder. The mixture was then pressed into thin pellets using a manual press and subjected to full-range scanning (4000–400 cm^−1^), performing a total of 64 scans at a resolution of 4 cm^−1^, with measurements taken at 25 °C. Finally, the recorded data were used to generate the Fourier Transform Infrared (FTIR) spectral profile.

### 2.5. Intrinsic Fluorescence Spectroscopy

Intrinsic fluorescence spectroscopy was employed to assess the tertiary structure of camel milk protein [26]. The protein was prepared in a phosphate-buffered saline (PBS) solution at pH 7.0, resulting in a sample concentration of 1.0 mg/mL. After being allowed to stand at a constant temperature for 2 h, it was ensured that the fluorescent probe reacted adequately with the protein molecule. Fluorescence emission spectra were measured within the range of 300–500 nm under an excitation wavelength of 295 nm and with slit widths set at 5.0 nm.

### 2.6. Observation of Protein Microstructure

The microstructure of protein particles was examined using a scanning electron microscope (SEM). The casein sample underwent freeze-drying and dehydration, followed by fixation on double-sided conductive adhesive tape to enhance conductivity through platinum coating. The microstructural analysis was conducted at an accelerated voltage of 15.0 kV with a magnification of ×12,000 using the Hitachi, Osaka, Japan, SU8010 SEM.

### 2.7. Analysis of Thermodynamic Properties of Proteins

Differential Scanning Calorimetry (DSC) (TA Instruments, New Castle, DE, USA, DSC2500) was employed to assess the thermal stability of casein. Approximately 5–10 mg of the sample was placed in a sealed aluminum dish and introduced into the equipment chamber at room temperature. Nitrogen gas, flowing at a rate of 40 mL/min, was utilized to maintain the local environment around the sample during analysis. The samples were subjected to scanning for 15 min at a heating rate of 10 °C/min within a temperature range from 0 °C to 150 °C. The initial (To, °C), peak (Tp, °C), and final denaturation temperatures (Td, °C), along with enthalpy changes associated with denaturation (ΔH, J/g), were recorded using an empty sealed crucible as reference material. Data obtained from this analysis were processed utilizing general analytical software provided with the DSC instrument.

### 2.8. Surface Hydrophobicity Measurement (Ho)

Following the methodology outlined by Yazdi Sr. et al. [27], 1-anilino-naphthalene-8-sulfonic acid (ANS) was employed as the hydrophobic fluorescent probe to determine Ho in treated casein samples. A stock solution containing 0.1% *w*/*v* casein was prepared by dissolving the sample in a pH 7.0 phosphate buffer solution, then subsequently diluted continuously with the same buffer until achieving final concentrations ranging from 0.004% to 0.02% *w*/*v*. An equal volume of ANS solution (20 µL; concentration: 8.0 mM in pH 7.0 phosphate buffer) was added to each diluted casein solution, totaling up to 4.0 mL per sample, for fluorescence measurement purposes using an LS55 fluorescence spectrometer (PerkinElmer Co., Springfield, IL, USA); fluorescence intensity (FI) readings were taken at the excitation wavelength of 390 nm and the emission wavelength of 470 nm. The initial slope derived from plotting FI against the protein concentration—calculated via linear regression analysis—served as an index for assessing surface hydrophobicity.

### 2.9. Preparation of TGase Enzyme Crosslinked Gel

For the gelation experiment, a casein gel was prepared using a 10% (*w*/*v*) casein solution in a 10 mm phosphate buffer at pH 7.0. The casein dispersion was injected into a glass mold with an inner diameter of 2.3 cm, covered with aluminum foil, and placed in a water bath, where it was heated from 25 °C to 95 °C and maintained at 95 °C for 30 min. Following this heating period, the casein gel within the glass mold was immediately cooled under running water and subsequently stored at 4 °C for 12 h to enhance and stabilize its network structure during cooling. The resulting cylindrical gel was utilized to analyze its water retention capacity (WHC), mechanical properties, and dynamic shear rheological characteristics.

### 2.10. Gel Water Retention

Each protein gel sample (2.0 g) underwent centrifugation at 4 °C with a speed of 10,000× *g* for 15 min. Subsequently, the supernatant containing free water released by loose structural units or unbound water components was carefully removed. The weights of the centrifuge tubes containing the gels were recorded before and immediately after centrifugation. Thus, WHC can be expressed as the ratio of the weight of the gel post centrifugation to its initial weight:

WHC (%) = (W_2_ − W_0_)/(W_1_ − W_0_) × 100 (%)

where W_2_ represents the weight (g) of centrifuge tubes containing gelatinous protein after centrifugation; W_1_ denotes their weight before centrifugation; and W_0_ indicates the weight of an empty centrifuge tube (g).

### 2.11. Texture Characteristics

The mechanical properties of the gel were assessed through uniaxial compression tests utilizing a TA-XT2i texture analyzer (Brookfield, CT3, New York, NY, USA), which was equipped with a 35 mm diameter cylindrical plate and a 12.0 mm diameter probe (P0.5R). A cylindrical protein gel (height: 1.0 cm; diameter: 2.3 cm) was compressed to 30% of its original height at a cross-head speed of 0.3 mm/s for a duration of 5 s. The data obtained were recorded and analyzed to derive the following parameters: hardness (maximum peak force during the initial compression cycle), elasticity (the height achieved during the second compression divided by the original compression distance), and cohesion (the ratio of the positive force area during the second compression to that during the first compression). Subsequently, chewability was calculated as hardness × cohesion × elasticity.

### 2.12. Dynamic Shear Rheological Properties

Dynamic shear rheological properties were measured using a rotating rheometer equipped with a temperature-controlled Peltier system (HAAKE MARS60+iS50; Thermo Fisher Scientific, Waltham, MA USA; Sichuan Jiede Trading Co., Ltd., Chengdu, China). Within the linear viscoelastic range, the constant angular frequency was maintained at 10 s^−1^, while the strain was set at 0.5%. A volume of 2.3 mL of protein gel was loaded onto the lower platen, followed by lowering an upper parallel platen (PP-50 probe; diameter: 50 mm) to establish contact with the sample at a gap of 1.0 mm. The temperature was increased from 25 °C to 95 °C at a heating rate of 2 °C/min, held steady at this temperature for an additional period of 30 min, and subsequently cooled back down to room temperature at a cooling rate also set at 2 °C/min. Silicone oil was applied to exposed areas in order to prevent sample desiccation throughout heating processes. During both heating and cooling cycles, the energy storage modulus (G′) and loss modulus (G″) for each sample were continuously monitored. The gel point was identified as the point where the elastic modulus (G′) and viscous modulus (G″) intersect, or where the loss tangent (tanδ = G″/G′) equals 1. Additionally, oscillation tests employing frequency sweeps ranging from 0.01 Hz to 100 Hz were performed under conditions maintaining strain levels fixed at one percent.

### 2.13. Statistical Analysis

Experiments were conducted in triplicate. Statistical analysis was subjected to one-way analysis of variance using SPSS 26.0 software (IBM SPSS Statistics for Windows, Version 26.0, IBM Corp., Armonk, NY, USA). Results were expressed as means ± SD, *p* < 0.05.

## 3. Results and Discussion

### 3.1. Sodium Dodecyl Sulfate Polyacrylamide Gel Electrophoresis (SDS-PAGE)

**Figure 1:**
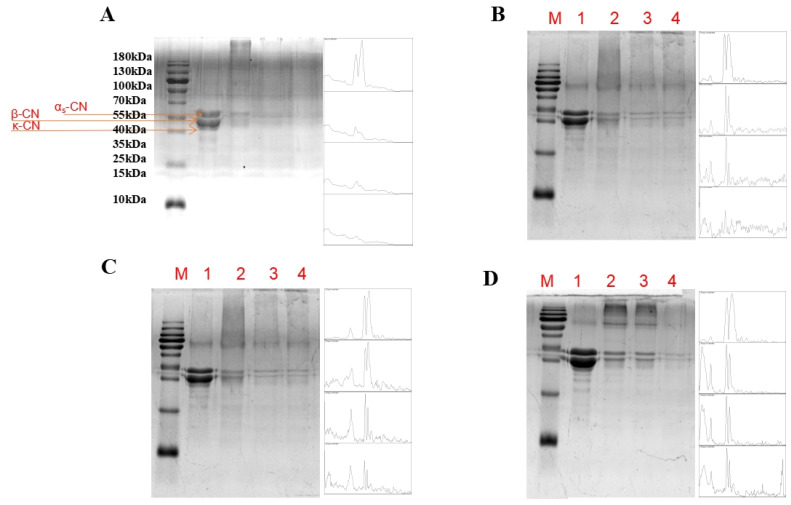
SDS-PAGE analysis of camel protein under various treatments. Note: (**A**) 25 °C, (**B**) 70 °C, (**C**) 80 °C, (**D**) 90 °C. Lane M represents the standard marker, while lanes 1–4 correspond to enzyme additions of 0%, 0.5%, 1%, and 1.5%, respectively.

The components of camel protein can be distinctly analyzed through SDS-PAGE, particularly focusing on the middle molecular weight range of the electrophoretic profile, which spans approximately 20–40 kDa. This range encompasses α-CN, β-CN, and κ-CN, with the intensity of the electrophoretic bands indicating that camel protein contains the highest proportion of β-CN. Followed by heat and enzymatic treatments, casein exhibited degradation to a certain extent (Figure 1). The grayscale of the α_S_-casein and β-casein spectra progressively lightened with increasing levels of enzyme supplementation. Chun-Chi Chen et al. used ultrasonic-assisted transglutaminase to investigate the crosslinking effects of αs-casein, β-casein, and κ-casein and found that with the increase in TGase, the spectral bands of αs-casein, β-casein, and κ-casein gradually became lighter [28]. Concurrently, heat treatment induced denaturation and aggregation of casein molecules, leading to the appearance of high-molecular-weight aggregate bands or broadened bands in the gel. This feature was particularly evident when treated with 0.5% TGase enzyme at 90 °C, resulting in the emergence of large molecular weight bands, indicative of the formation of new protein polymers through enzymatic crosslinking; under the addition of 1% and 1.5% TGase, the band strength of casein monomer gradually diminished. In a study by Bulca S et al. (2022) [29], which investigated the crosslinking reaction of microbial transglutaminase (TGase) in camel milk yogurt, SDS-PAGE analysis revealed that the crosslinking reaction between lysine and glutamine amino acids, catalyzed by TGase, resulted in the formation of macromolecular products. The intensity of the monomer bands decreased with the increasing concentration of TGase, correlating with the formation of aggregates.

### 3.2. Fourier Transform Infrared Spectroscopy (FTIR)

Infrared spectroscopy serves as a crucial method for determining the relative content of protein secondary structures. By analyzing the absorption peaks of the amide I and amide III bands, the secondary structure of proteins were quantitatively assessed. The infrared spectra underwent baseline correction, followed by Gaussian deconvolution and second-derivative fitting. Subsequently, the ratio of each secondary structure was calculated based on the peak area, thereby revealing the proportions and dynamic changes in various structural elements.

**Figure 2:**
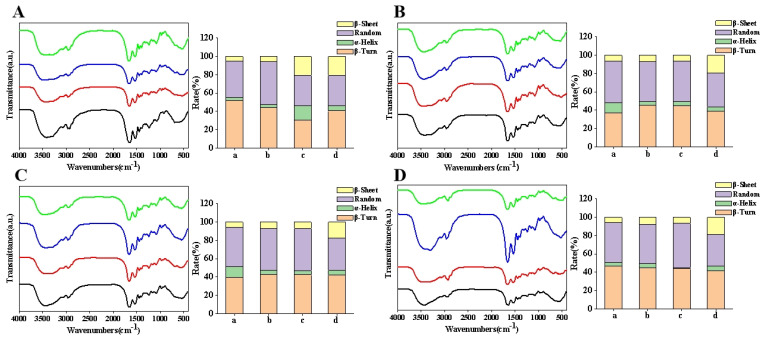
FTIR spectra of samples subjected to various treatments. Note: (**A**–**D**) correspond to the Fourier infrared spectrum of camel casein samples treated with different enzymes at temperatures of 25 °C, 70 °C, 80 °C, and 90 °C, respectively. Enzyme addition: **--** 0%, **--** 0.5%, **--** 1%, **--** 1.5%; a–d refer to the proportions of each secondary structure at enzyme addition levels of 0–1.5%, respectively: β-Sheet, Random, α-Helix, β-Turn.

After enzymatic treatment, the absorption peak of caseinamide I shifted to a higher frequency (Figure 2A), and the crosslinked structure enhanced intermolecular interactions, resulting in a more compact molecular arrangement. TGase facilitates the transition from loose structures (such as random curling) to ordered structures (such as beta-folding), thereby increasing molecular rigidity and stability [30]. After heat treatment, the FTIR spectra of casein exhibited wide and strong absorption peaks around 3400 cm^−1^, indicating the presence of a significant number of intramolecular or intermolecular hydrogen bonds. The heat treatment disrupted the spatial structure of casein, leading to the breaking of intramolecular hydrogen bonds. Following treatment at 90 °C for 30 min, the caseinamide I band was redshifted towards lower wavenumbers, suggesting that hydrophobic amino acid residues were exposed and new intermolecular hydrogen bonds were formed. Simultaneously, heat treatment significantly altered the proportion of the secondary structure of casein: the content of random curls increased, while the β-fold content initially rose and then declined, indicating that some of the ordered structures were converted into random curls.

In Figure 2B,C, the α-helix content gradually increased with the addition of the enzyme, indicating the enhanced structural stability of the protein. Thermal denaturation of the protein leads to the gradual breaking of hydrogen bonds within the α-helix, resulting in the uncoiling of the helix. This process allows the intermolecular β-fold structure to easily transform into a β-turn structure, while some β-turn structures may also convert into random coil structures [31]. The increase in α-helix content following enzymatic treatment is associated with protein denaturation and the gradual formation of aggregates. As illustrated in Figure 2D, at 90 °C with a 30 min heat treatment, the α-helix content is lowest with 1% enzyme addition, while the random coil content is the highest. The α-helix structure is typically linked to specific protein functions, including the active sites of enzymes and binding regions of receptors. A reduction in α-helix content may lead to alterations in the biological activity of the protein, while an increase in random coil content may enhance the protein’s flexibility and accessibility, thereby affecting its interactions with other molecules.

### 3.3. Endogenous Fluorescence Spectrum

**Figure 3:**
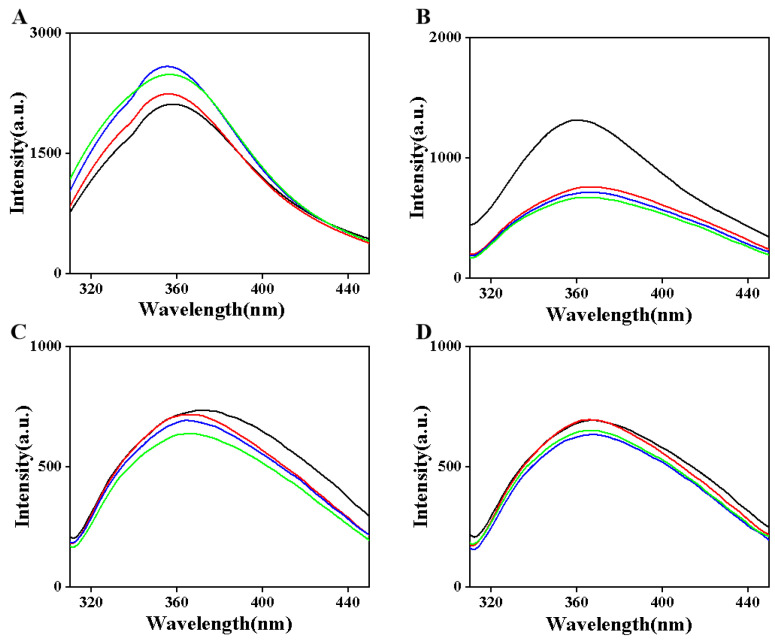
The internal fluorescence spectra of samples treated with different excitation wavelength of 295 nm. Note: (**A**–**D**) correspond to the endogenous fluorescence spectra of camel casein samples treated with different enzymes at temperatures of 25 °C, 70 °C, 80 °C, and 90 °C, respectively; Enzyme addition: **--** 0%, **--** 0.5%, **--** 1%, **--** 1.5%.

Certain amino acid residues in proteins, such as tryptophan, tyrosine, and phenylalanine, exhibit natural fluorescence properties, with tryptophan displaying the strongest fluorescence intensity and contributing significantly to the overall fluorescence. Endogenous fluorescence spectroscopy measures the fluorescence emission of these amino acids at specific wavelengths to extract structural information about proteins. As illustrated in Figure 3A, an increase in the fluorescence peak value indicates a change in the microenvironment of the fluorescence chromophore, leading to an enhanced fluorescence quantum yield. A left shift in the fluorescence spectrum, characterized by the emission wavelength moving towards shorter wavelengths, typically signifies a decrease in polarity or an increase in hydrophobicity surrounding the chromophore. This phenomenon may result from the crosslinking reaction of casein catalyzed by TGase enzymes, which tightens the protein’s tertiary structure, causing amino acid residues that were previously exposed to the aqueous environment to become sequestered within the interior. Consequently, this leads to a blue shift in the fluorescence emission wavelength [32]. In instances of heat treatment alone, a decrease in the fluorescence peak value suggests a reduction in overall fluorescence intensity. This may be attributed to the destabilization of the tertiary structure of casein due to heat treatment, resulting in the exposure of more fluorescence chromophores that were initially buried within the protein to the external polar environment, thereby causing fluorescence quenching and a subsequent decrease in peak value [33]. Simultaneously, a redshift in the emission wavelength indicates an increase in polarity or a decrease in hydrophobicity, confirming a significant alteration in the protein’s tertiary structure.

As the amount of TGase enzyme addition increases, the fluorescence peak gradually decreases and exhibits a redshift (Figure 3B). This phenomenon may be attributed to the combined effects of TGase enzyme activity and heat treatment, which significantly alter the tertiary structure of casein. The introduction of the enzyme loosens the protein structure, thereby exposing the fluorescence chromophore to the external polar environment, leading to fluorescence quenching. Following heat treatment at 80 °C (Figure 3C), an increase in TGase enzyme addition results in a decrease in the fluorescence peak value and a blue shift, indicating that the enzyme-promoted crosslinking reaction is enhanced. Consequently, the protein structure becomes more compact, reducing the interaction between the chromophore and its environment and diminishing the likelihood of fluorescence quenching. After heat treatment at 90 °C (Figure 3D), the addition of 0.5% TGase enzyme causes the fluorescence emission wavelength to shift to shorter wavelengths, suggesting a decrease in polarity around the initial chromophore or an increase in hydrophobicity. As the amount of TG enzyme continues to increase, the fluorescence peak value decreases, while the emission wavelength shifts to longer wavelengths. This may be due to over-crosslinking or the emergence of a new quenching mechanism, which makes the environment surrounding the chromophore more hydrophilic, resulting in a redshift.

### 3.4. Determination of Protein Microstructure

**Figure 4:**
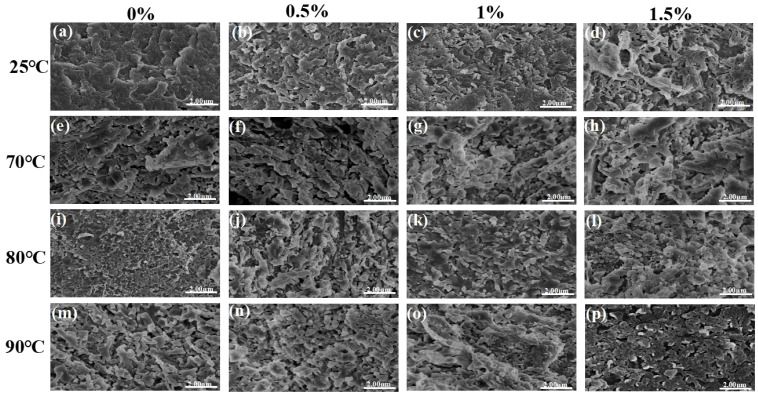
Scanning electron microscope (SEM) images of casein after heat treatment and enzyme treatment (×12,000). (**a**–**p**): Morphological structural changes of casein under varying temperatures (rows: 25 °C, 70 °C, 80 °C, 90 °C) and enzyme concentrations (columns: 0%, 0.5%, 1%, 1.5%).

The microstructure of camel casein protein samples was analyzed by using SEM. As illustrated in the figure, the original casein exhibits a blocky and compact structure (Figure 4a). Following heat and enzyme treatments, the casein gradually evolves into a network structure characterized by small pore sizes and tight crosslinks (Figure 4b–p). After heat treatment (Figure 4e,i,m), casein displays a distinct “pore” structure. This transformation is attributed to the disruption of hydrogen bonds and salt bridges caused by heat, leading to the breakdown of the “jacket” layer structure of κ-casein. This results in an irregular micelle surface and an increase in particle size. Elevated temperatures may also induce the cleavage and recombination of chemical bonds within casein molecules, thereby affecting the tertiary structure of the proteins. For instance, cracks emerge in the originally flat casein particles, and the aggregation or precipitation resulting from heat treatment manifests as larger block-like structures or aggregates in SEM images [34]. When the sample with 0.5% TGase enzyme addition was incubated at 90 °C, a more dense and uniform network structure was observed (Figure 4l). As the enzyme concentration increased, the aggregation behavior among protein molecules intensified, resulting in a tightly crosslinked, “fluffy” internal structure. Similar findings have been reported in studies involving other animal proteins, such as whey protein and egg albumin [35,36]. The combination of TGase enzyme and heat treatment significantly impacts the morphological stability of casein. On one hand, crosslinking enhances the structural integrity of casein, rendering it more resistant to deformation induced by heat treatment. Conversely, excessive heat treatment can compromise the crosslinked structure, resulting in notable morphological alterations. Scanning Electron Microscopy (SEM) images clearly illustrate the differences in casein’s morphological stability under varying treatment conditions.

### 3.5. Analysis of Thermodynamic Properties of Casein Proteins

After enzymatic treatment, the thermal transition temperature decreased, while the enthalpy value increased. This phenomenon may be due to the reason that TGase enzymes facilitate the crosslinking of casein molecules, potentially enhancing overall structural flexibility [37]. Furthermore, the treatment may alter the spatial structure of the protein, which reduces spatial obstruction and consequently lowers the thermal transition temperature. Changes in the water distribution and state also amplify the plasticizing effect of water. Heat treatment disrupts the secondary and tertiary structures of proteins, leading to aggregation or depolymerization, which further contributes to the reduction of the thermal transition temperature.

**Figure 5:**
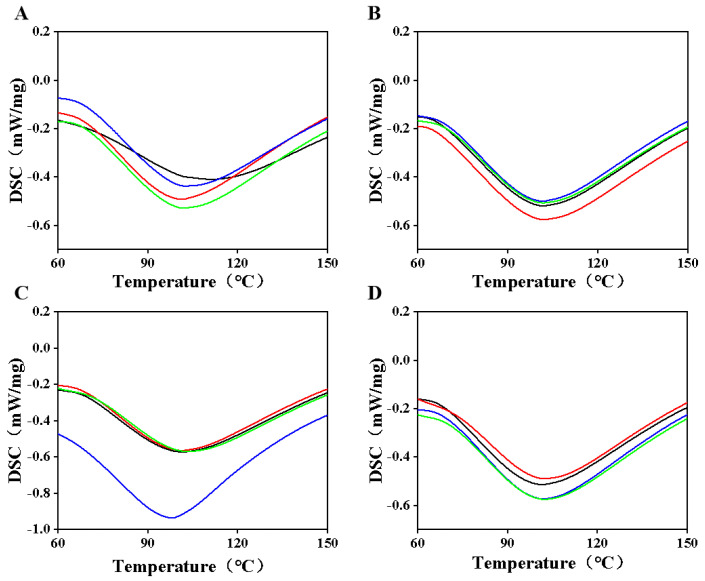
Differential Scanning Calorimetry (DSC) profiles following heat treatment at 25 °C, 70 °C, 80 °C, and 90 °C, as well as enzyme treatment at concentrations of 0%, 0.5%, 1%, and 1.5%. Note: (**A**–**D**) correspond to the DSC maps of camel casein samples treated with various enzymes at the specified temperatures; Enzyme addition: **--** 0%, **--** 0.5%, **--** 1%, **--** 1.5%.

Figure 5B shows that with 0.5% TGase enzyme addition, both the thermal transition temperature and enthalpy values of camel casein protein increased. The TGase enzyme facilitates the crosslinking reaction between casein molecules, resulting in a more compact and stable structure, thereby enhancing thermal stability and shifting the characteristic peak towards higher temperatures. As the amount of TGase enzyme increases, protein new interactions are formed (such as hydrogen bonding and hydrophobic interactions), and it may absorb more energy, leading to an increase in enthalpy value. Following heat treatment at 80 °C, partly protein molecules unfolded or became loose. Upon the addition of TGase enzyme, the thermal transition temperature initially decreased and then increased with the enzyme concentration (Figure 5C). The 0.5% TGase enzyme may further depolymerize the partially unfolded protein molecules, forming an unstable crosslinked structure that renders the protein structure irregular and loose, thus reducing the thermal transition temperature. As the addition of TG enzyme increases, camel casein forms large-molecular-weight aggregates, and the crosslinking reaction is gradually enhanced. After heat treatment at 90 °C, the enthalpy value of camel casein protein firstly increased and then decreased with the increasing of the enzyme concentration (Figure 5D). This may be attributed to structural changes in certain proteins caused by the 90 °C treatment, while the addition of TGase enzymes promotes the formation of new interactions with other molecules, necessitating more energy to disrupt these interactions, resulting in an increase in enthalpy value. However, when the TGase enzyme is added in excess (1.5%), over-crosslinking occurs, causing the protein structure to become rigid and tight, leading to a decrease in enthalpy value [38]. Excess TGase enzyme may also contribute to the aggregation and precipitation of camel casein protein, further reducing the enthalpy value.

### 3.6. Surface Hydrophobicity (Ho)

**Figure 6:**
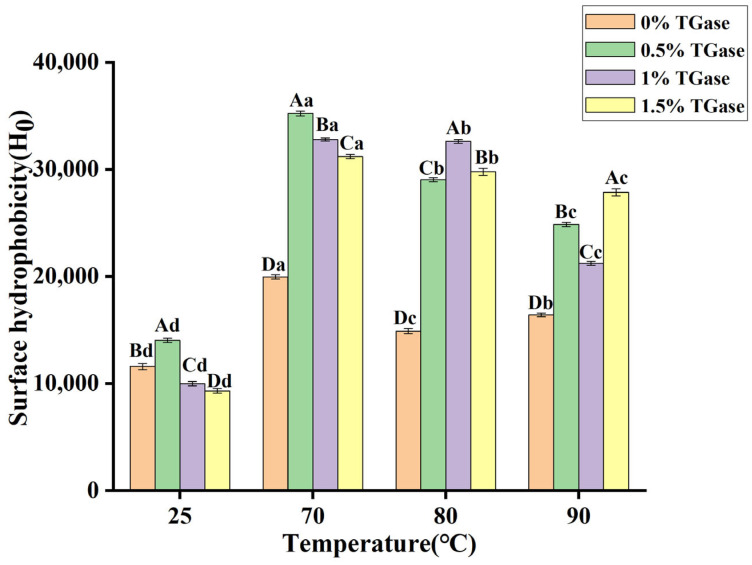
Surface hydrophobicity (Ho) of camel casein after heat treatment (25 °C, 70 °C, 80 °C, and 90 °C) and enzyme treatment (0%, 0.5%, 1%, and 1.5%). Note: bars with different capital letters (A–D) indicate significant differences in casein treated at the same temperature but with different enzyme levels, while bars with different lowercase letters (a–d) indicate significant differences in casein treated at the same enzyme levels but at different temperatures (*p* < 0.05).

The addition of 0.5% TGase significantly improved the surface hydrophobicity of casein (Figure 6). This enzyme catalyzes acyl group transfer reactions between and within protein molecules, leading to the formation of ε-(γ-glutamyl) lysine covalent bonds between casein molecules. This crosslinking effect alters the structure of casein, exposing some hydrophobic groups that were previously hidden within the interior to the surface, thereby increasing surface hydrophobicity. However, excessive enzyme addition may lead to over-crosslinking, resulting in aggregation and precipitation of casein. In such cases, the effective surface area of casein may decrease, consequently reducing its surface hydrophobicity. The surface hydrophobicity of casein increased significantly following both heat treatment and enzyme treatment. Heat treatment induces changes in the tertiary structure of casein, exposing hydrophobic amino acid residues that were originally concealed within the protein. Under heating conditions, the spatial structure of the protein is disrupted, allowing the previously folded hydrophobic regions to come into contact with the environment more easily, thus increasing surface hydrophobicity [39]. However, the surface hydrophobicity significantly decreased with the increase in heat treatment temperature while maintaining a constant enzyme amount. This phenomenon may be attributed to the elevated temperatures enhancing the thermal motion of casein molecules, which facilitates aggregation among protein molecules. Consequently, the hydrophobic regions within the aggregates become less exposed to the environment, leading to a reduction in surface hydrophobicity [40].

### 3.7. Gel Water Retention

**Figure 7:**
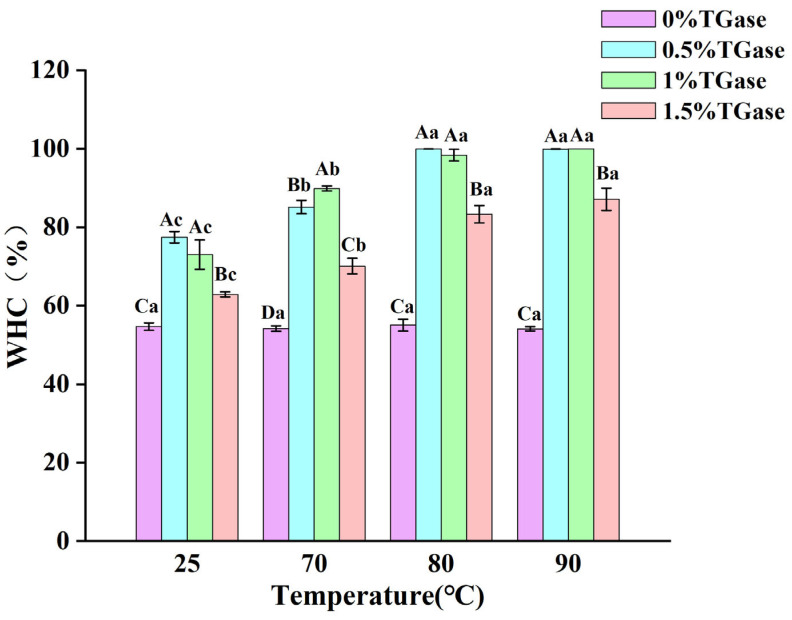
Water retention of camel casein gel during heat treatment (25 °C, 70 °C, 80 °C, and 90 °C) and enzyme treatment (0%, 0.5%, 1%, and 1.5%). Note: bars with different capital letters (A–D) indicate significant differences in casein treated at the same temperature but at different enzyme levels, while bars with different lowercase letters (a–c) indicate significant differences in casein treated at the same enzyme levels but at different temperatures (*p* < 0.05).

Water retention is an important index for measuring the ability of a gel to retain water, reflecting the interaction between protein and water molecules. The results indicated that the water retention of the gel was not significantly affected by heat treatment alone (Figure 7). This suggests that casein does not effectively disrupt the hydrophilic groups of proteins or alter their spatial conformation during heat treatment, thereby maintaining the binding ability of proteins and water molecules at a relatively stable level [41]. In contrast, enzymatic treatment significantly enhanced the water retention of casein protein gel. The addition of TGase enzymes may alter the molecular structure of casein, exposing hydrophilic groups that were previously concealed within the molecule. These hydrophilic groups can form additional hydrogen bonds and other interactions with water molecules. Following enzymatic treatment, some amino acid residues may become exposed, allowing their polar side chains to bind with water molecules, thus significantly improving water retention [42]. At temperatures of 25 °C, 70 °C, 80 °C, and 90 °C, the water retention capacity of the gel supplemented with 1.5% TGase was significantly lower compared to that of gels supplemented with 0.5% and 1.0% TGase. This phenomenon may be related to the excessive crosslinking of casein caused by the excessive addition of TGase. The increase in TG enzyme concentration leads to the increase in pores in protein gel, and thus no tight network structure is formed to bind water in the gel system, resulting in the decrease in water retention.

### 3.8. Mechanical Properties

It is challenging for camel protein to form a measurable gel structure under single heat treatment or single enzyme treatment. This difficulty may be attributed to the significant differences in amino acid composition and spatial structure of camel casein compared to other common milk casein sources [43,44]. The action of TGase on proteins exhibits a certain substrate specificity, primarily targeting protein regions with specific amino acid sequences or structures. The unique structure of camel protein may hinder its ability to provide suitable substrate sites for TGase enzymes, thereby limiting the effective catalytic crosslinking reaction between casein molecules and further affecting gel formation. The hardness of the gel increases significantly with rising temperature when the enzyme amount is kept constant (Table 1). As the temperature rises, the thermal motion of casein molecules intensifies, leading to a higher frequency of intermolecular collisions, which promotes the formation of additional crosslinking bonds and enhances the likelihood of crosslinking catalyzed by TGase enzymes. Additionally, some amino acid residues that are typically buried within the protein may become exposed at elevated temperatures, making them accessible targets for TGase and further facilitating the crosslinking reaction, resulting in increased gel hardness. However, at a specific heat treatment temperature, increasing the enzyme concentration can lead to a decrease in gel hardness. This phenomenon may be due to an excessive amount of TGase causing casein to over-aggregate, resulting in the formation of larger aggregates. These aggregates may not be uniformly distributed within the gel, potentially causing local stress concentrations that lead to voids and defects inside the gel, thereby reducing its compactness and hardness, and ultimately diminishing the overall mechanical properties [45].

### 3.9. Rheological Properties

#### 3.9.1. Dynamic Rheological Characteristics

The results indicate that throughout all heating durations, both the energy storage modulus (G′) and the loss modulus (G″) of the gel exhibit a tendency to stabilize as the temperature increases. This stabilization suggests that the gelation process is nearing completion, effectively characterizing the transformation of the sample from a low-viscosity liquid (liquid-like) to a high-strength gel (solid-like) [46]. Following the addition of 0.5% TG enzyme, the gel point decreased from 34.90 °C to 29.90 °C. Notably, the loss modulus prior to the gel point was greater than the energy storage modulus. This observation indicates that TGase facilitates the crosslinking reaction between casein molecules, thereby accelerating protein aggregation and crosslinking, enhancing intermolecular interactions, and promoting gelation at lower temperatures [47]. When the gel is not fully formed, the system remains closer to a liquid state, where viscous flow predominates. This results in significant energy dissipation, leading to a high loss modulus. As the amount of enzyme added increases, the gel point disappears, and G′ consistently exceeds G″, indicating a gradual transition of the system to a more elastic state. The increased concentration of TGase may further enhance the degree of crosslinking, resulting in a more compact gel structure.

**Figure 8:**
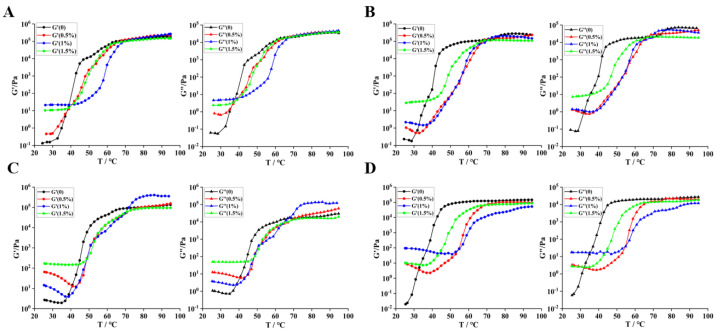
Illustrates the continuous scanning of the oscillating temperature of samples subjected to various treatments. Note: (**A**–**D**) correspond to the chromatograms of camel casein samples treated with different enzymes at temperatures of 25 °C, 70 °C, 80 °C, and 90 °C, respectively. Enzyme addition: **--** 0%, **--** 0.5%, **--** 1%, **--** 1.5%.

**Figure 9:**
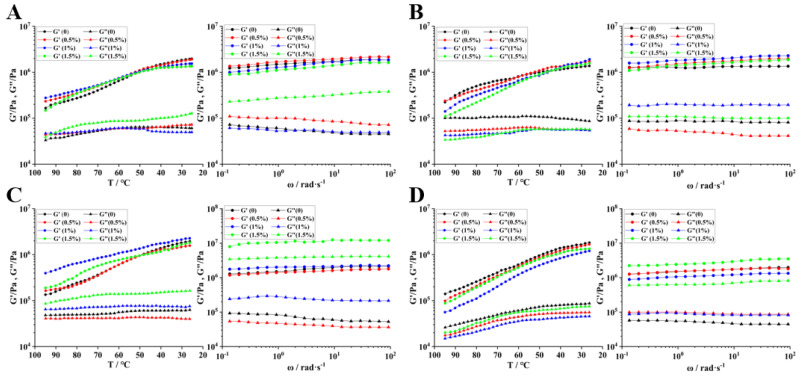
Oscillating cooling continuous scanning and frequency scanning of samples with different treatments. Note: (**A**–**D**) refer to the chromatograms of camel casein samples treated with different enzymes at 25 °C, 70 °C, 80 °C, and 90 °C respectively. Enzyme addition: **--** 0%, **--** 0.5%, **--** 1%, **--** 1.5%.

Under heat treatment, the peaks of the G′ after varying temperature treatments were recorded as 18.7 × 10^4^ Pa, 27.8 × 10^4^ Pa, 12.4 × 10^4^ Pa, and 14.16 × 10^4^ Pa at 25 °C, 70 °C, 80 °C, and 90 °C, respectively. At 70 °C, the casein gel exhibited significant elastic deformation. Figure 8B illustrates that in the following treatment at 70 °C with the addition of enzyme under different concentrations, both G′ and G″ are decreased, indicating that the combined effects of temperature and enzymes altered the structure of the casein gel. The increase in temperature intensified the movement of protein molecules, while the enzyme facilitated intermolecular reactions, collectively leading to the structural instability of the gel. At 80 °C, under the influence of 1% TGase enzyme, G′ reached 38.9 × 10^4^ Pa (Figure 8C), suggesting that the gel structure was relatively optimized at this point, possessing an adequate degree of crosslinking to ensure elasticity while preventing excessive crosslinking that could hinder molecular movement. Heating can cause natural proteins to unfold, exposing polar groups. This process leads to a more ordered water structure and promotes the aggregation of hydrophobic regions, thereby enhancing the hydrophobic interactions between casein and the heavy chain. Ultimately, this results in a stronger and more elastic final gel structure [48]. At 90 °C, G′ and G″ are initially decreased then increased with the increasing addition of enzymes (Figure 9D). This phenomenon may attribute to high temperatures diminishing the stability of protein molecules, while enzymatic activity further exacerbates this instability, resulting in a decrease in modulus. The subsequent rise in G′ and G″ with increased enzyme addition indicates that the enzyme facilitates the reformation or reinforcement of the gel structure. Enzymes catalyze new crosslinking reactions or modify the arrangement of protein molecules, thereby tightening the gel structure and gradually restoring and enhancing elasticity and viscosity [49].

#### 3.9.2. Shear Rheological Properties

During the oscillating cooling continuous scanning process, the gradual decrease in temperature slows the movement of protein molecules, promoting the formation of hydrogen bonds and hydrophobic interactions, thereby enhancing the elasticity of the gel. Following the addition of TGase enzyme, as intermolecular interactions intensified, the degree of crosslinking within the gel increased, resulting in a rise in G′ as the temperature decreased, which signifies an enhancement in the gel’s elasticity [50]. At this stage, G′ is significantly greater than G″, and G′ remains relatively stable or changes slowly, indicating that the gel predominantly exhibits elastic behavior characterized by a high elastic modulus and low viscous loss.

In the higher frequency region, the G′ curve is flatter (Figure 9B,C), indicating that the elasticity of the gel remains relatively stable at high frequencies. In contrast, the G″ curve exhibits varying slopes across different frequency ranges, reflecting the frequency-dependent properties of viscosity. The protein gel maintains its elastic structure at high frequencies due to robust crosslinked networks formed by disulfide bonds and hydrogen bonds, resulting in minimal changes in G′ with frequency (Figure 9D). Following the combination of heat treatment and enzyme treatment (Figure 9B–D), the G″ curve becomes relatively flat, suggesting the presence of balanced hydrogen bonds, van der Waals forces, or other non-covalent interactions that moderately impede the relative motion of molecules, leading to stable energy dissipation [51]. These results indicate that the addition of TGase and temperature changes play a crucial role in regulating the rheological properties of camel protein gels.

## 4. Conclusions

The effects of TGase enzyme combined with heat treatment on the structure and gel properties of camel casein were investigated. When 0.5% TG enzyme was added at 90 °C, new large-molecular-weight protein aggregates emerged, and the degree of protein aggregation and crosslinking intensified with increased TGase enzyme addition. The absorption peak of the amide I band shifted to a higher frequency, indicating a transformation from a loose, disordered secondary structure of casein to a more ordered structure. The TGase enzyme catalyzes the crosslinking reaction of casein, resulting in a more compact tertiary structure of the protein. Following heat treatment and enzyme treatment, casein formed a network structure characterized by smaller pore sizes and tighter crosslinking; this network structure became more compact and uniform with the addition of 0.5% TGase enzyme at 90 °C. The introduction of TGase enzyme lowered the heat transition temperature and increased the enthalpy change, while significantly enhancing the surface hydrophobicity of casein. Heat treatment did not have a significant effect on the water retention of the gel; however, enzyme treatment markedly improved the water retention of the gel, and gel hardness increased significantly with rising temperature. Treatment with 0.5% TGase enzyme significantly reduced the gel point, promoted gelation, improved the viscoelastic properties of the gel, and facilitated the transition of the gel to an elastic state.

## Figures and Tables

**Table 1:** Effects of temperature and enzyme addition on the texture of camel casein gel.

Temperature (°C)	Enzyme Addition (%)	Hardness (gf)	Elasticity (gf)	Chewability (gf)	Adhesion (gf)	Cohesion	Resilience
**25**	**0**	0	0	0	0	0	0
	**0.5**	0	0	0	0	0	0
	**1**	0	0	0	0	0	0
	**1.5**	0	0	0	0	0	0
**70**	**0**	0	0	0	0	0	0
	**0.5**	7.63 ± 0.67 ^Ab^	0.40 ± 0.52 ^ABa^	2.31 ± 3.10 ^Ab^	5.19 ± 0.72 ^Bb^	0.68 ± 0.12 ^Bb^	0.59 ± 0.14 ^Ba^
	**1**	7.96 ± 0.61 ^Ac^	0.22 ± 0.06 ^Bc^	1.51 ± 0.44 ^Ab^	6.95 ± 0.31 ^Ac^	0.87 ± 0.03 ^Aa^	0.72 ± 0.08 ^ABa^
	**1.5**	4.71 ± 0.23 ^Bc^	0.93 ± 0.07 ^Aa^	3.09 ± 0.41 ^Ab^	3.32 ± 0.24 ^Cb^	0.71 ± 0.07 ^ABa^	0.84 ± 0.02 ^Aa^
**80**	**0**	0	0	0	0	0	0
	**0.5**	18.53 ± 2.77 ^Aa^	0.69 ± 0.04 ^Aa^	11.92 ± 1.87 ^Aa^	17.25 ± 1.69 ^Aa^	0.94 ± 0.05 ^Aa^	0.74 ± 0.05 ^Aa^
	**1**	12.67 ± 2.30 ^Bb^	0.57 ± 0.06 ^Bb^	6.51 ± 1.05 ^Bb^	11.46 ± 0.79 ^Bb^	0.92 ± 0.12 ^Aa^	0.82 ± 0.06 ^Aa^
	**1.5**	10.86 ± 0.51 ^Bb^	0.49 ± 0.03 ^Bb^	4.74 ± 0.84 ^Bab^	9.72 ± 1.23 ^Ba^	0.89 ± 0.10 ^Aa^	0.75 ± 0.11 ^Aa^
**90**	**0**	0	0	0	0	0	0
	**0.5**	21.10 ± 0.46 ^Aa^	0.68 ± 0.02 ^Aa^	11.87 ± 1.04 ^Aa^	17.50 ± 0.95 ^Aa^	0.83 ± 0.03 ^Aab^	0.76 ± 0.06 ^Aa^
	**1**	18.69 ± 0.67 ^Ba^	0.71 ± 0.01 ^Aa^	11.89 ± 0.65 ^Aa^	14.75 ± 2.75 ^ABa^	0.90 ± 0.04 ^Aab^	0.75 ± 0.06 ^Aa^
	**1.5**	12.56 ± 0.38 ^Ca^	0.53 ± 0.07 ^Bb^	5.91 ± 1.64 ^Ba^	11.07 ± 1.52 ^Ba^	0.88 ± 0.15 ^Aa^	0.71 ± 0.09 ^Aa^

Note: different capital letters (A–C) indicate significant differences in casein treated at the same temperature but at different enzyme levels, while different lowercase letters (a–c) indicate significant differences in casein treated at the same enzyme levels but at different temperatures (*p* < 0.05).

## Data Availability

The original contributions presented in this study are included in the article; further inquiries can be directed to the corresponding author.
